# Moxonidine Increases Uptake of Oxidised Low-Density Lipoprotein in Cultured Vascular Smooth Muscle Cells and Inhibits Atherosclerosis in Apolipoprotein E-Deficient Mice

**DOI:** 10.3390/ijms24043857

**Published:** 2023-02-14

**Authors:** Yutang Wang, Dinh Tam Nguyen, Jack Anesi, Ahmed Alramahi, Paul K. Witting, Zhonglin Chai, Abdul Waheed Khan, Jason Kelly, Kate M. Denton, Jonathan Golledge

**Affiliations:** 1 Discipline of Life Science, Institute of Innovation, Science and Sustainability, Federation University Australia, Ballarat, VIC 3350, Australia; 2 Molecular Biomedicine Theme, School of Medical Sciences, Faculty of Medicine and Health, Charles Perkins Centre, The University of Sydney, Sydney, NSW 2006, Australia; 3 Department of Diabetes, Central Clinical School, Monash University, Melbourne, VIC 3004, Australia; 4 Fiona Elsey Cancer Research Institute, Ballarat, VIC 3350, Australia; 5 Department of Physiology, Monash University, Melbourne, VIC 3800, Australia; 6 Cardiovascular Disease Program, Monash Biomedicine Discovery Institute, Monash University, Melbourne, VIC 3800, Australia; 7 Queensland Research Centre for Peripheral Vascular Disease, College of Medicine and Dentistry, James Cook University, Townsville, QLD 4811, Australia; 8 Department of Vascular and Endovascular Surgery, The Townsville University Hospital, Townsville, QLD 4814, Australia

**Keywords:** moxonidine, atherosclerosis, inflammation, cell migration

## Abstract

This study aimed to investigate the effect of the sympatholytic drug moxonidine on atherosclerosis. The effects of moxonidine on oxidised low-density lipoprotein (LDL) uptake, inflammatory gene expression and cellular migration were investigated in vitro in cultured vascular smooth muscle cells (VSMCs). The effect of moxonidine on atherosclerosis was measured by examining aortic arch Sudan IV staining and quantifying the intima-to-media ratio of the left common carotid artery in apolipoprotein E-deficient (ApoE^−/−^) mice infused with angiotensin II. The levels of circulating lipid hydroperoxides in mouse plasma were measured by ferrous oxidation-xylenol orange assay. Moxonidine administration increased oxidised LDL uptake by VSMCs via activation of α2 adrenoceptors. Moxonidine increased the expression of LDL receptors and the lipid efflux transporter ABCG1. Moxonidine inhibited mRNA expression of inflammatory genes and increased VSMC migration. Moxonidine administration to ApoE^−/−^ mice (18 mg/kg/day) decreased atherosclerosis formation in the aortic arch and left common carotid artery, associated with increased plasma lipid hydroperoxide levels. In conclusion, moxonidine inhibited atherosclerosis in ApoE^−/−^ mice, which was accompanied by an increase in oxidised LDL uptake by VSMCs, VSMC migration, ABCG1 expression in VSMCs and lipid hydroperoxide levels in the plasma.

## 1. Introduction

Atherosclerosis is a progressive disease of large- and medium-sized arteries in which dyslipidaemia plays an instrumental role in the disease pathogenesis [1,2,3]. When an atherosclerotic plaque develops an unstable phenotype, it is prone to rupture, which is thought to be the mechanism of acute cardiovascular events such as myocardial infarction and stroke [1]. Despite tremendous advances in the medical management of atherosclerosis through lifestyle changes and pharmacological control of low-density lipoprotein (LDL) cholesterol and triglyceride levels, blood pressure, diabetes and thrombosis, atherosclerosis-associated morbidity and mortality remains the number one health threat in most countries [1]. This highlights the need to develop innovative therapeutic strategies to prevent atherosclerotic plaque formation and rupture.

The sympathetic nervous system governs the “fight-or-flight” response [4]. The main overall end effect of the system is to prepare the body for physical activity, while also affecting many organs (e.g., kidney [5]) and physiological functions (e.g., metabolism [6]). The sympathetic nervous system may affect atherosclerosis. For example, stress, which leads to the activation of the sympathetic nervous system [7,8], is a critical risk factor for atherogenesis [9]. The class of β blocker drugs inhibit sympathetic nervous activity, but their adverse effects on lipoprotein levels and glucose metabolism prohibit their use as bespoke inhibitors of atherosclerosis [10]; some new-generation β blockers have some favourable effects on lipoprotein and glucose metabolism, although their effect on atherosclerosis prevention remains unclear [10]. Renal sympathetic denervation has been reported to inhibit atherosclerosis in mice [11], although this is not without controversy [12]. Furthermore, renal sympathetic denervation may permanently damage renal nerves and could result in renal artery stenosis in some patients [13]. Therefore, there is a need to investigate the effect of the sympathetic nerve system on atherosclerosis with other non-invasive approaches such as the use of sympatholytic drugs.

It is unknown whether moxonidine (a sympatholytic drug) inhibits atherosclerosis, although the drug has some activities that may be linked to an anti-atherogenic role, such as the inhibition of inflammation [14,15]. This study aimed to investigate the effect of moxonidine on atherosclerosis.

Angiotensin II can promote atherosclerosis formation [16]. On the other hand, blocking angiotensin II formation by angiotensin-converting enzyme (ACE) inhibitors can inhibit atherogenesis [17] and prevent cardiovascular events (e.g., myocardial infarction, stroke and cardiovascular mortality) [18]. Therefore, the angiotensin-infusion-induced atherosclerosis model is a clinically relevant model to study atherosclerosis. In addition, angiotensin stimulates the sympathetic nervous system [19], and overactivation of the sympathetic nervous system is seen in many conditions associated with atherosclerosis such as myocardial infarction [20] and stroke [21]. Therefore, this study chose angiotensin II infusion as the model of atherosclerosis to investigate the effect of moxonidine (a sympatholytic drug) on atherosclerosis.

Oxidised LDL plays a role in foam cell formation [22], a hallmark of atherosclerosis. This study also aimed to investigate the effect of moxonidine on oxidised LDL uptake by cultured vascular smooth muscle cells (VSMCs), as recent evidence suggests that VSMCs may be a major pathway for foam cell formation [23]. For example, 50% of foam cells within human coronary artery lesions were derived from VSMCs, as indicated by the expression of the smooth muscle cell-specific marker SM α-actin [24]. In addition, in apolipoprotein E-deficient (ApoE^−/−^) mice supplemented with a Western diet for 6 weeks, 70% of foam cells in aortic arch lesions were derived from VSMCs as indicated by the expression of smooth muscle cell-specific fluorescent proteins [25].

## 2. Results

### 2.1. Moxonidine Increased Oxidised LDL Uptake via α2 Adrenoceptors by VSMCs In Vitro

**Figure 1:**
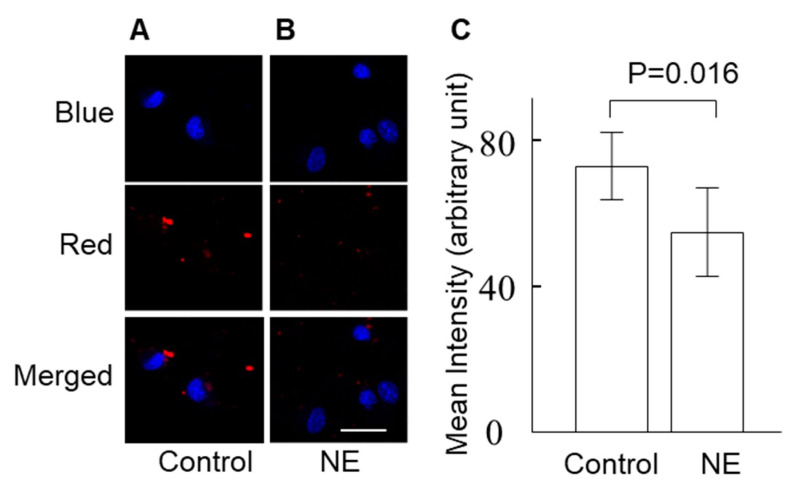
Norepinephrine (NE) increased oxidised low-density lipoprotein (LDL) uptake in vascular smooth muscle cells (VSMCs). VSMCs were incubated with NE (0.1 μM) or phosphate-buffered saline (PBS, control) for 30 min, and then the cells were treated with Dil-labelled oxidised LDL (25 μg/L) for 4 h. After staining the nucleus with Hoechst, the intracellular red fluorescence (Dil-labelled oxidised LDL) and blue fluorescence (nucleus) were imaged via a confocal microscope. (**A**,**B**) Representative images of oxidised LDL (red channel), nucleus (blue channel) and merged channels of cells in PBS-incubated (**A**) and NE-incubated cells (**B**). Scale bar = 40 µm. (**C)** The mean intensity of intracellular Dil-labelled oxidised LDL fluorescence. The mean intensity of Dil-labelled oxidised LDL fluorescence (red) in the cells was analysed using ImageJ. The difference between the groups was analysed using the Mann–Whitney U test. Data represent mean ± SD; N = 8.

**Figure 2:**
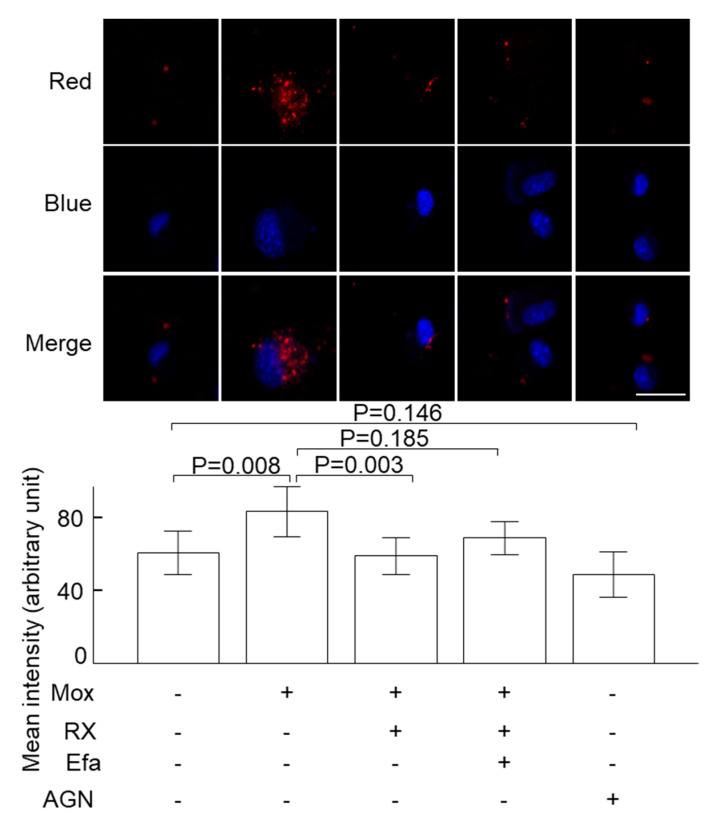
The effect of moxonidine on oxidised LDL uptake by VSMCs. VSMCs were incubated with RX821002 (10 μM, α2 adrenoceptor inhibitor), efaroxan (10 μM, α2 adrenoceptor and I1 receptor inhibitor) or AGN192403 (100 μM, I1 receptor activator) for 30 min and moxonidine (10 μM) or phosphate-buffered saline (PBS, control) was then added to the cells. After another 2 h, Dil-labelled oxidised LDL (25 μg/L) was added to all the cells and the red fluorescence (engulfed Dil-labelled oxidised LDL) inside of each cell was visualised by a confocal microscope after 4 h. The mean fluorescence intensity of the cells in the dish was calculated using ImageJ. The differences among groups were analysed using Kruskal–Wallis one-way ANOVA followed by Bonferroni’s post hoc tests. Data represent mean ± SD; N = 8. Scale bar = 40 µm. AGN, AGN192403; Efa, efaroxan; Mox, moxonidine; RX, RX821002.

**Figure 3:**
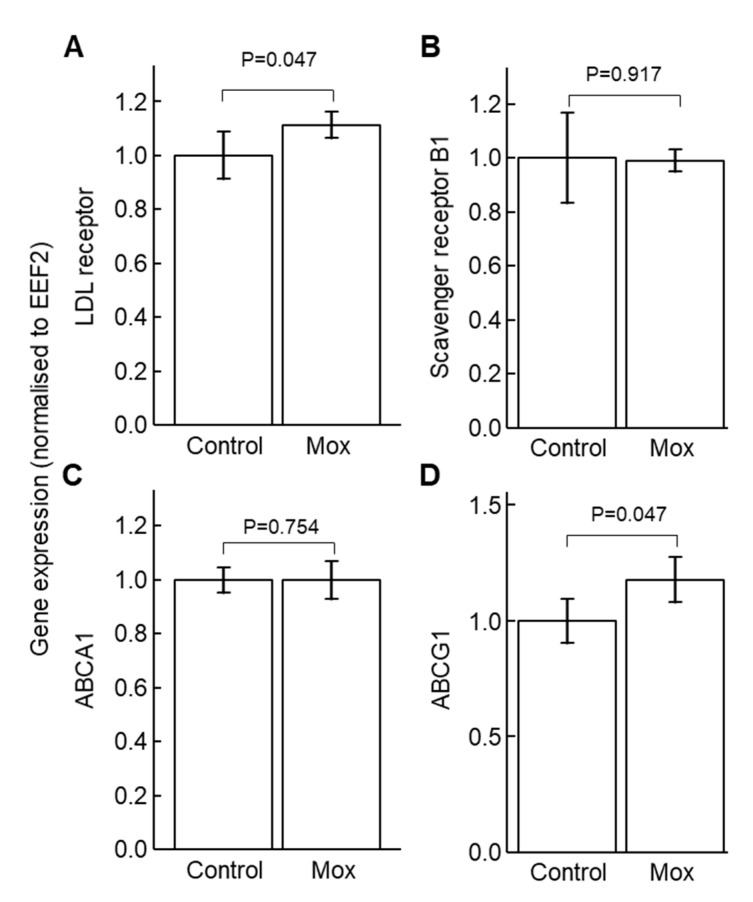
The effect of moxonidine on mRNA expression of genes related to lipid uptake and efflux. VSMCs were incubated with moxonidine (10 μM) or phosphate-buffered saline (PBS, control) for 2 h and the cells were then incubated with oxidised LDL (10 μg/L) for an additional 4 h. mRNA was extracted and the expressions of the LDL receptor (**A**), scavenger receptor B1 (**B**), ABCA1 (**C**) and ABCG1 (**D**) were quantified via quantitative PCR. The difference was analysed by Mann–Whitney U test. Data represent mean fold-change (vs. control) ± SD; N = 5. ABCA1, ATP binding cassette subfamily A member 1; ABCG1, ATP binding cassette subfamily G member 1; EEF2, eukaryotic elongation factor; LDL, low-density lipoprotein; Mox, moxonidine; VSMC, vascular smooth muscle cells.

Incubation of cultured VSMCs with the sympathetic activator norepinephrine (0.1 µM) decreased the uptake of oxidised LDL (Figure 1). Consistently, incubation of the cells with the sympatholytic drug moxonidine increased the uptake of oxidised LDL as indicated by the microscopic analysis (Figure 2) and total cholesterol levels (Figure S1). Moxonidine is an agonist for both α_2_ adrenoceptors and imidazoline I_1_ receptors [26,27,28]. To study this moxonidine-mediated increase in oxidised LDL uptake further, a series of inhibitor and activator studies were performed. Notably, oxidised LDL uptake was reversed by co-incubation with RX821002 (α_2_ adrenoceptor inhibitor, 10 µM) or efaroxan (inhibitor for both α_2_ adrenoceptor and I_1_ receptor, 10 µM) with the drugs employed at final doses known to specifically target their respective receptor [29,30]. By contrast, the uptake of oxidised LDL was not enhanced by activation of the I_1_ receptor-specific activator AGN192403 [31] (100 µM) (Figure 2). Together, these observations suggest that moxonidine increased the uptake of oxidised LDL through the activation of α_2_ adrenoceptors. Gene expression studies showed that in the presence of oxidised LDL, moxonidine (10 µM) increased gene expression of the LDL receptor but not the scavenger receptor (Figure 3A,B). In addition, moxonidine (10 µM) increased the expression of the lipid efflux gene ABCG1, but not ABCA1 (Figure 3C,D).

### 2.2. Moxonidine Inhibited Inflammatory Gene Expression in Lipopolysaccharide-Treated VSMCs and Endothelial Cells In Vitro

**Figure 4:**
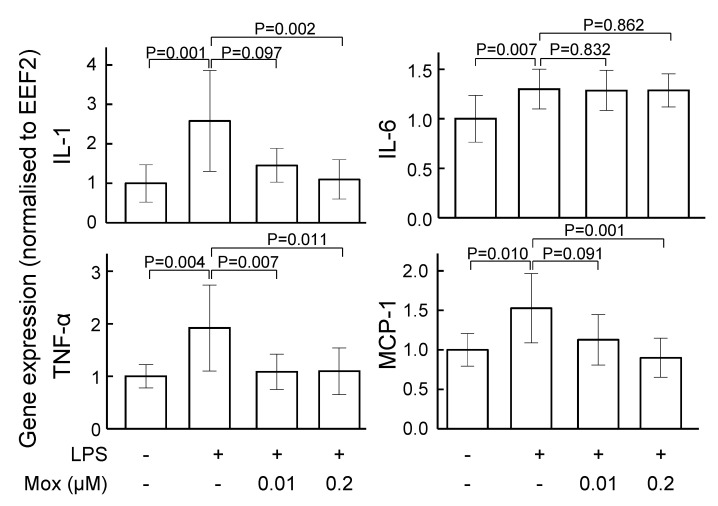
The effect of moxonidine on mRNA expression of inflammatory markers in VSMCs. Cells were incubated with lipopolysaccharide for 2 h in the absence or presence of moxonidine (0.01 or 0.2 μM). Then, mRNA was isolated and the expressions of IL1, IL-6, MCP-1 and TNF-α were quantified via quantitative PCR. The difference was analysed by Kruskal–Wallis one-way ANOVA followed by Bonferroni’s post hoc test. Data represent mean fold-change (vs. control) ± SD; N = 7–12. EEF2, eukaryotic elongation factor; IL, interleukin; LPS, lipopolysaccharide; MCP-1, monocyte chemoattractant protein-1; Mox, moxonidine; TNF-α, tumour necrosis factor-α; VSMC, vascular smooth muscle cells.

Incubation of VSMCs with lipopolysaccharide increased inflammatory gene expression (Figure 4). Moxonidine (0.2 µM) significantly decreased mRNA expression of interleukin 1 (IL-1), monocyte chemoattractant protein-1 (MCP-1) and tumour necrosis factor-α (TNF-α) (Figure 4). However, moxonidine did not affect the mRNA expression of inflammatory markers in cultured endothelial cells (Figure S2).

### 2.3. Moxonidine Enhanced VSMC Migration Without Affecting VSMC Proliferation In Vitro

**Figure 5:**
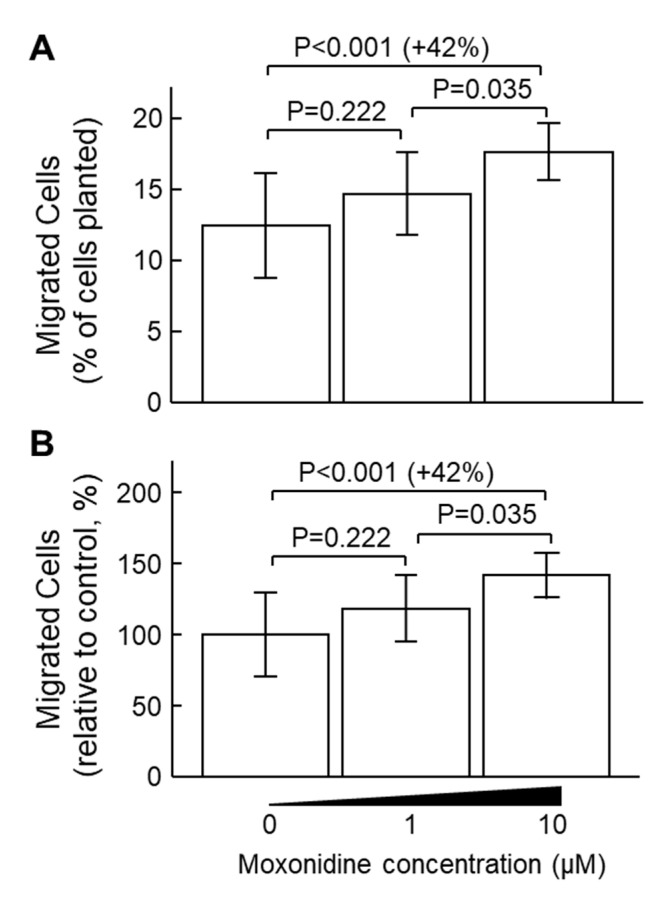
Effect of moxonidine on cellular migration of vascular smooth muscle cells (VSMCs). VSMCs in serum-free DMEM media were placed in the upper chamber of cell migration chambers, with or without moxonidine at the indicated concentrations. The lower chamber contained 150 μL of DMEM medium containing 20% foetal bovine serum. After 48 h incubation, the migrated cells in the lower chamber were stained and the fluorescence (an indicator of cell numbers) was measured (excitation/emission = 530/590 nm). (**A**) Migratory cells were represented as a percentage of the total number of cells planted in the upper chamber. (**B**) Migratory cell numbers relative to the untreated controls (0 μM moxonidine). The difference among the group was analysed using Kruskal–Wallis one-way ANOVA followed by Bonferroni’s post hoc tests. Data represent mean ± SD; N = 11–12 per group.

Incubation with moxonidine (10 µM) stimulated the migration of VSMCs by 42% (Figure 5). However, incubation with moxonidine at concentrations of 0.015, 0.15, 1.5, 15 and 150 µM for 24 h did not affect cell proliferation as assessed by the MTS assay (Figure S3). Corroborating results were obtained when VSMC proliferation was assessed by the trypan blue method (Figure S4).

### 2.4. Moxonidine Decreased Atherosclerosis in Angiotensin-Infused ApoE^−/−^ Mice

**Figure 6:**
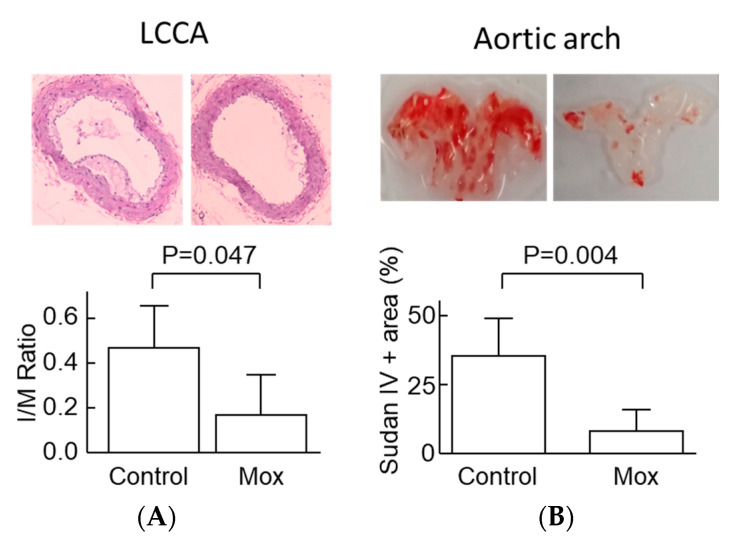
Effect of moxonidine on atherosclerosis in apolipoprotein E-deficient mice. (**A**) The left common carotid artery was stained with the H&E method. The ratio of the lesion area over the medial area (I/M ratio) was calculated. Magnification = 10×. (**B**) The aortic arch was stained with Sudan IV to visualise the lipid in the lumen surface of the aortic arch. The difference between the two groups was analysed by the Mann–Whitney U test. Error bars represent SD; N = 6 per group. H&E, hematoxylin and eosin; LCCA, left common carotid artery; Mox, moxonidine.

**Figure 7:**
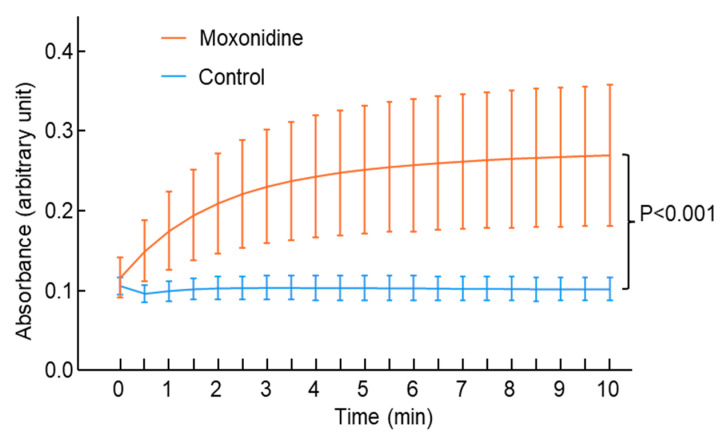
Effect of moxonidine administration on plasma lipid peroxide levels. Mice were treated with moxonidine (18 mg/kg body weight/day) via drinking water until the end of the experiment, while the control mice received normal drinking water only. Three days after the initiation of moxonidine administration, angiotensin II was subcutaneously infused into all the mice for 28 days to induce atherosclerosis. Mice were culled at the end of the angiotensin II infusion and plasma was collected. Lipid peroxide levels in the plasma were measured every 30 s. Higher absorbance = higher lipid peroxide levels. The difference in plasma lipid peroxide levels between the two groups was analysed using multiple linear regression: dependent variable = absorbance and independent variables = groups and time. Data represent mean ± standard error; N = 5 per group.

To investigate the effect of moxonidine on atherosclerosis directly, we used angiotensin II infusion for 4 weeks to induce atherosclerosis formation in ApoE^−/−^ mice. Compared with the control (without moxonidine), moxonidine administration (18 mg/kg/day) decreased atherosclerosis formation in the aortic arch as assessed by Sudan IV staining (Figure 6) as well as in the left common carotid artery as assessed by morphometry analysis (intimal/medial area ratio, Figure 6). Surprisingly, moxonidine administration increased lipid peroxide levels in the plasma of the mice (Figure 7). Association studies showed that higher lipid peroxide levels were associated with the presence of atherosclerosis [32], suggesting that lipid peroxide may be proatherogenic. Therefore, the plasma lipid peroxide data appeared in disagreement with the in vivo finding that moxonidine administration decreased atherosclerosis.

## 3. Discussion

This study found that the sympatholytic drug moxonidine increased the uptake of oxidised LDL, stimulated mRNA expression of the LDL receptor and the ABCG1 transporter, enhanced cell migration (but not proliferation) and inhibited inflammatory gene expression in vitro in cultured VSMCs. In addition, moxonidine administration in ApoE^−/−^ mice inhibited atherosclerosis formation induced by angiotensin II infusion.

Moxonidine is a sympatholytic drug with blood pressure lowering properties [26]. Biological activity for moxonidine involves the deactivation of the sympathetic nervous system with parallel decreases in the plasma norepinephrine level [28,33]. Herein, this study found, for the first time, that moxonidine decreased atherosclerosis formation in a mouse model of atherosclerosis that was induced by subcutaneous infusion of angiotensin II. This mouse model of atherosclerosis is clinically relevant [16], as it mimics increased angiotensin signalling in patients with atherosclerosis and cardiovascular disease [18,20,21].

At four weeks (the end of the experiment), the body weight of the mice was similar between the moxonidine and control groups (Figure S5), and so was the body weight change during the experiment (Figure S6). Administration of moxonidine for 4 weeks did not change organ weight, including of the kidney, spleen and heart (Figure S7). In addition, the mice treated with moxonidine did not show apparent signs of illness during the experiment (e.g., diarrhoea, abnormal gait and abnormal breathing), suggesting that 4-week administration of moxonidine may not cause apparent toxicity. However, the toxicity associated with longer-term (>4 weeks) administration of moxonidine is unknown, as is the effect of moxonidine at the cerebral level. These questions need to be investigated in the future.

VSMCs in the intima have been considered beneficial for plaque stability, as VSMCs constitute the main cellular component of the protective fibrous cap within lesions and are responsible for synthesising extracellular matrix components that stabilise the cap [23,34]. Therefore, an increase in VSMC migration may be beneficial. The current study, for the first time, showed that incubation with moxonidine stimulated VSMC migration that potentially contributes to plaque stabilisation.

Atherosclerosis is an inflammatory disease [35,36]. Pro-inflammatory cytokines and chemokines (e.g., MCP-1 and TNF-α) play a key role in the initiation and progression of atherosclerosis [37]. The current study showed that moxonidine inhibited the lipopolysaccharide-induced increase in TNF-α expression. This is consistent with a previous report which demonstrated that moxonidine treatment in hypertensive postmenopausal women decreased circulating TNF-α levels [14]. We also showed for the first time that moxonidine decreased MCP-1 mRNA expression. Our results and those from the literature [14,15] support the notion that moxonidine has an anti-inflammatory effect, which might play an important role in mediating the anti-atherosclerotic effect of moxonidine.

Endothelial dysfunction is a major contributor to atherogenesis, and enhanced inflammation is a key mechanism underlying endothelial dysfunction [38,39]. Our results showed that treatment of endothelial cells with moxonidine did not affect the gene expression of inflammatory markers, including IL-1, IL-6, MCP-1 and TNF-α. This suggests that the effect of moxonidine may be cell type specific, i.e., moxonidine may target VSMCs rather than endothelial cells. This seems in agreement with the sympathetic innervation pattern of the vasculature. It is well known that arteries are innervated with sympathetic nerves [40] and the nerve endings are distributed in the smooth muscle layer but not the endothelial layer [41]. Therefore, VSMCs, rather than endothelial cells, may be the key target of moxonidine in blood vessels. However, the current study did not investigate the effect of moxonidine on endothelial function and this needs to be investigated in the future.

High levels of LDL are a risk factor for atherosclerosis formation. In localised intimal microenvironments, where antioxidant defences have been overwhelmed, LDL can be oxidised to initiate atherogenesis [42]. Although moxonidine does not affect plasma LDL levels [27,43] or subclass pattern nor oxidation susceptibility [43], it may inhibit oxidation of LDL in the inflammatory subendothelial space, as moxonidine inhibited inflammatory gene expression in VSMCs.

Oxidised LDL plays a significant role in atherogenesis. In the inflammatory intimal microenvironments, LDL can be oxidised and taken up by macrophages, leading to foam cell formation [44,45]. VSMCs are recently recognised as an important source of foam cells in atherosclerosis lesions [23]. For example, VSMC-derived foam cells accounted for 50% of foam cells within advanced human coronary artery lesions [24] and accounted for 70% of foams cells in the aortic arch lesions in apolipoprotein E-deficient (ApoE^−/−^) mice [25].

Our results showed that moxonidine increased oxidised LDL uptake by activation of α2-adrenoceptors, which was associated with an increase in the mRNA expression of the LDL receptor. In addition, plasma lipid peroxide levels in the mice were increased after moxonidine treatment. These results suggest that moxonidine may be proatherogenic. However, this interpretation contradicted the in vivo finding that moxonidine decreased atherosclerosis, which is supported by the anti-inflammatory effect of moxonidine. The contradicting observations suggest that an alternative explanation may be needed.

The functions of the recently discovered VSMC-derived foam cells are poorly understood. These cells are believed to promote atherogenesis [24,25], because they showed a decrease in the lipid efflux transporter ABCA1 [24,25]. Moxonidine did not affect ABCA1 gene expression. Interestingly, it increased the expression of ABCG1, another key lipid efflux transporter. This is the first report that showed that gene expression of lipid efflux transporters could be increased in lipid-laden VSMCs. This suggests that moxonidine may change the phenotype of the VSMC-derived foam cells from accumulating oxidised LDL inside the cells to effluxing oxidised LDL out of the blood vessel to circulation for detoxification and elimination by the liver. This hypothesis fits the in vivo finding that moxonidine administration decreased atherosclerosis. In addition, this oxidised LDL efflux hypothesis is supported by the finding that moxonidine administration increased plasma lipid peroxide levels. Moreover, increased VSMC migration by moxonidine may also support this hypothesis, as VSMC migration to a favourable location could facilitate both uptake of oxidised LDL and its efflux back to circulation. This hypothesis seems to harmonise the observed contradicting results, but it is speculative in nature and needs to be investigated in the future.

**Figure 8:**
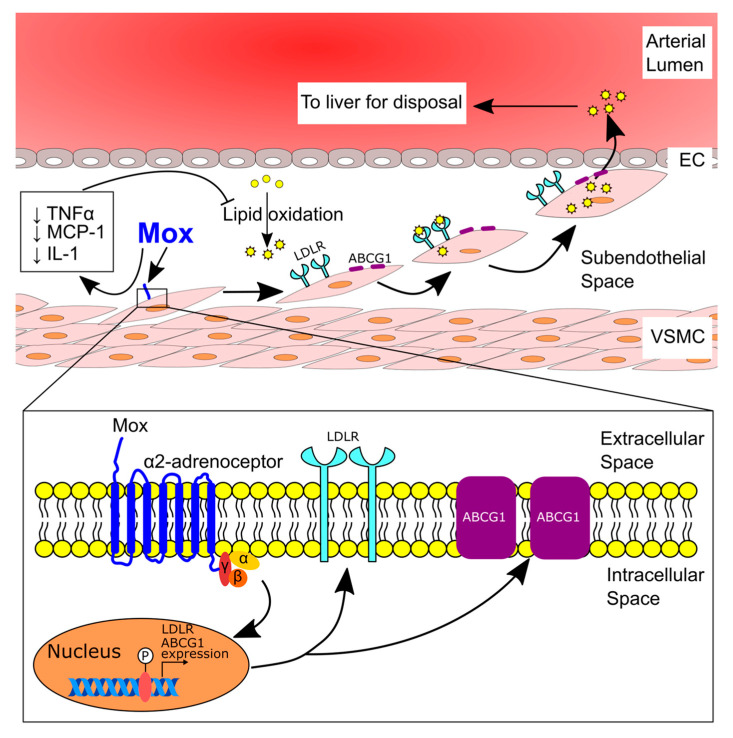
Hypothesis: proposed mechanism underlying moxonidine-induced inhibition of atherosclerosis. Moxonidine decreases the expression of inflammatory genes (e.g., TNF-α), which inhibit the oxidation of LDL. Moxonidine enhances VSMC migration. These VSMCs then migrate to a location that could facilitate both oxidised LDL uptake via the LDL receptor and its efflux back to circulation via the ABCG1 transporter for detoxification and elimination by the liver. Thus, moxonidine may inhibit atherosclerosis by inhibiting inflammation and promoting oxidised LDL clearance from the atherosclerotic plaque. ABCG1, ATP binding cassette subfamily G member 1; EC, endothelial cell; IL, interleukin; LDL, low-density lipoprotein; LDLR, low-density lipoprotein receptor; MCP-1, monocyte chemoattractant protein-1; Mox, moxonidine; TNF-α, tumour necrosis factor-α; VSMC, vascular smooth muscle cell.

Therefore, we propose the following mechanism to explain the atherosclerosis inhibitory effect of moxonidine: moxonidine inhibits the expression of inflammatory genes (e.g., IL-1, MCP-1 and TNF-α), which subsequently may inhibit the oxidation of LDL (Figure 8). In addition, moxonidine increases the uptake of oxidised LDL by VSMCs via activation of α_2_-adrenoceptors. As moxonidine increases VSMC migration this may result in relocation of VSMCs to facilitate the uptake of oxidised LDL via the LDL receptor and efflux back to circulation via the ABCG1 transporter for detoxification and elimination by reverse cholesterol transport (Figure 8). This hypothesis warrants further investigation in the future.

## 4. Materials and Methods

### 4.1. Animals

Male ApoE^−/−^ mice (3 months old) were purchased from the Animal Resources Centre, Perth, Australia. All experiments were conducted in a temperature-controlled animal house (21 ± 1 °C) under a 12:12 h light–dark cycle, and mice were given standard chow and water ad libitum.

### 4.2. Experimental Protocol

Twenty ApoE^−/−^ mice were randomised into two groups according to their age and body weight (N = 10 per group): the control group and the moxonidine-treated group. The mice in the control group received plain drinking water, whereas mice in the moxonidine treatment group received moxonidine via drinking water (18 mg/kg/day) [46]. Three days after the initiation of the moxonidine administration, angiotensin II was administered subcutaneously to all the mice via a micro-osmotic pump (Model 2004, ALZET, Cupertino, CA, USA) at a rate of 1 µg/kg body weight/min for 28 days to induce atherosclerosis [12,16,47].

Four mice from each of the two groups died of aortic rupture, which is common in angiotensin II-infused mice [47,48]. The quality of the aortic tissue from these dead mice prevented them from inclusion in atherosclerosis assessment. Therefore, atherosclerosis was only assessed in the surviving animals (N = 6 per group).

### 4.3. Quantification of Atherosclerotic Lesion Area

Atherosclerosis in the aortic arch was quantified by en face staining as described previously [12]. Briefly, the aortic arch was opened longitudinally and pinned down on a wax-coated petri dish. Tissue samples were stained with 0.1% *w*/*v* Sudan IV for 10 min to identify areas of atherosclerosis. Sudan-IV-stained areas were quantified using ImageJ 1.53e and expressed as a percentage of the total aortic arch luminal surface area.

Atherosclerosis in the left common carotid artery was assessed using morphometry analysis [49]. In brief, formalin-fixed and paraffin-embedded left common carotid arteries were sectioned (5 μm thickness) starting from the labelled proximal end. The location where the first complete arterial structure (a circular structure) appeared was designated the location of 0 μm. Four serial arterial sections (at locations 0, 160, 320 and 480 μm) were subsequently obtained and stained with hematoxylin and eosin (H&E). Images of the stained sections were captured using a light microscope (Nikon, Tokyo, Japan). The area of the atherosclerotic lesion and medial area was quantified using Photoshop (Microsoft, version 22.0.0) and the corresponding intima-to-media area was calculated for each section. The average of the ratios from the 4 serial sections was assigned as the final measurement of atherosclerosis in that left common carotid artery.

### 4.4. Ferrous Oxidation-Xylenol Orange (FOX) Assay

A FOX assay was used to assess levels of lipid hydroperoxides in mouse plasma [50]. Briefly, 50 µL FOX solution (250 µM ammonium sulphate, 100 mM D-sorbitol and 125 µM xylenol orange) was added to 50 µL mouse plasma and the mixture was incubated at 20 °C. Absorbance was measured using a FLUOstar Omega reader (BMG LABTECH Pty. Ltd, Mornington, Australia) and a time-dependent change in output at 560 nm was determined at 30 s intervals with the linear rate of xylenol orange compared between different treatment groups.

### 4.5. Cell Culture

VSMCs were isolated from the mouse aorta [51] and cultured as previously described [52]. Briefly, VSMCs were cultured at 37 °C in Dulbecco’s modified Eagle medium (DMEM) supplemented with 10% *v*/*v* foetal bovine serum, 100 U/mL penicillin and 100 μg/mL streptomycin in a cell culture incubator containing 5% CO_2_. The cells were split with 0.05% *w*/*v* trypsin when they reached 80% confluency and subcultured for further passages.

Human aortic endothelial cells were purchased from Lonza Australia Pty Ltd. (Mount Waverley, Australia). These cells were cultured in EGM™-2 Endothelial Cell Growth Medium supplemented with growth factors required for culturing endothelial cells (Lonza Australia Pty Ltd.).

### 4.6. Confocal Microscopy for Oxidised LDL Uptake

VSMCs (5 × 10^5^ cells) in DMEM + 1% foetal bovine serum were seeded in MatTek confocal dishes. Following 24 h of subculture, cells were then incubated separately with 10 µL of RX821002 (α_2_ adrenoceptor inhibitor [29], final concentration = 10 µM), efaroxan (inhibitor for both α_2_ adrenoceptor and I_1_ receptor [30], final concentration = 10 µM) or AGN192403 (I_1_ receptor activator [31], final concentration = 100 µM). The cells were equilibrated further for 30 min at 37 °C, and then 10 µL of moxonidine was added to each of the confocal dishes (final concentration = 10 µM) and the cells were further incubated for 2 h. Dil-labelled oxidised LDL (Thermo Fisher Scientific Australia Pty Ltd., Scoresby, Australia, final concentration = 25 µg/L) was added and the cells were incubated for another 4 h away from light. Next, 2 µL of Hoechst was added to the cell dishes for 10 min to stain the nuclei and the cells were washed with phosphate-buffered saline before the culture medium was replaced by a phenol-free DMEM. The red fluorescence (engulfed Dil-labelled oxidised LDL) was imaged by a confocal microscope and the mean fluorescence intensity of each cell on the image was measured using ImageJ. Eight images were taken for each dish at random locations across the whole dish. The mean intracellular fluorescence intensity from these 8 images was calculated as the final intracellular fluorescence intensity for that dish.

### 4.7. Gene Expression Analysis

Effect of moxonidine on inflammatory gene expression in VSMCs: VSMCs (6 × 10^5^ cells per well) in 4 wells of 6-well plates were incubated with moxonidine (0, 0, 0.01 or 0.2 μM) for 12 h. Then, the cells were treated with lipopolysaccharide (0, 100, 100 or 100 ng/mL, respectively). After a further 2 h incubation, RNA was extracted using the TRI reagent (Merck, Bayswater, Australia).

Effect of moxonidine on the expression of genes related to lipid uptake and efflux: VSMCs (1 × 10^6^ cells per well) in 6-well plates were cultured for 24 h and then incubated with moxonidine (10 μM) or phosphate-buffered saline (PBS, control) for 2 h. Oxidised LDL (10 μg/L) was then added to the cells which were cultured for an additional 4 h. Next, mRNA was extracted using the TRI-reagent (Merck).

Effect of moxonidine on inflammatory gene expression in endothelial cells: cells (2.5 × 10^5^ cells per well) in 3 wells of 6-well plates were cultured for 48 h. Then, the cells were treated with moxonidine (0, 1 or 1 μM) for 2 h. Following this, the cells were treated with lipopolysaccharide (0, 100 or 100 ng/mL, respectively). After a further 2 h incubation, RNA was extracted using the TRI reagent (Merck).

The extracted RNA was reverse transcribed to cDNA using the High-Capacity Reverse Transcription Kit (Life Technologies, Carlsbad, CA, USA). Gene expression was assessed by quantitative PCR using SYBR reagents (Bioline Global Pty Ltd., Gregory Hills, Australia). Primer sets are outlined in Table S1. The cycling conditions were as follows: a hold at 95 °C for 2 min, followed by 40 cycles at 95 °C for 15 s, 58 °C for 20 s and 72 °C for 20 s. Relative gene expression was assessed using the 2^−ΔΔCt^ method [53]. Gene expression analysis was represented using relative gene expression compared with the control gene eukaryotic translation elongation factor 2 (EEF2) [12].

### 4.8. Migration Assay

Migration assay was conducted using a cell migration assay kit from Abcam (Cambridge, UK) according to the manufacturer’s instructions. In brief, 50 µL of VSMCs (50,000 cells) in a serum-free DMEM were added to the top chamber in addition to 50 µL of serum-free DMEM containing 0, 2 or 20 µM moxonidine. The final concentration of moxonidine in the top chamber was 0, 1 or 10 µM. The bottom chamber contained 150 µL of DMEM + 20 % of foetal bovine serum per well. The cells were incubated in a CO_2_ incubator at 37 °C for 48 h. The migrated cells in the lower chamber were washed and stained and the fluorescence (an indicator of cell numbers) was measured using a plate reader (excitation/emission = 530/590 nm).

### 4.9. Cell Proliferation

Cell proliferation was conducted using an MTS cell proliferation assay kit (Abcam) as previously described [51,54]. In brief, 200 µL of VSMCs (0.5 × 10^6^ cells/mL) was added to each well of a 96-well flat-bottom plate and kept at 37 °C in an incubator overnight. Next, 2 µL of moxonidine at different concentrations was added to give a final concentration of 0, 0.015, 0.15, 1.5, 15 or 150 µM. After the cells were incubated for 24 h, 20 µL of MTS reagent was added to each well and the cells were incubated for another 2 h. Finally, the absorbance was recorded using a plate reader at 520 nm.

Cell proliferation was also assessed using the trypan blue method [51,54]. In brief, 2 mL of VSMCs (5 × 10^4^ cells/mL) was placed in wells of 6-well plates and incubated in a 5% CO_2_ incubator at 37 °C for 24 h. Then, various concentrations of moxonidine were added to give a final concentration of 0.01, 0.1, 1 or 10 µM. After 24 h of incubation, the cells were trypsinised, stained with trypan blue and then counted using Countess Automated Cell Counter (Invitrogen, Waltham, MA, USA).

### 4.10. Total Cholesterol

VSMCs (5 × 10^4^ cells) were seeded in 96-well plates. Following 24 h of subculture, 2 µL of moxonidine (final concentration in the wells = 10 µM) or PBS was added, and the cells were further incubated for 2 h. Oxidised LDL (final concentration = 25 µg/L) was added to all the wells and the cells were incubated for another 4 h. The cells were washed and lysed and the supernatant was collected after centrifugation at 12,000 g for 10 min. Total cholesterol in the supernatant was then measured using a commercial kit from Abcam according to the manufacturer’s instructions [55].

### 4.11. Statistical Analyses

The difference between two groups was analysed using Mann–Whitney U test [56] and the difference among multiple groups was analysed using Kruskal–Wallis one-way AVOVA. The difference in plasma lipid peroxide levels between two groups (with or without moxonidine) was analysed using multiple linear regression [57,58]: dependent variable = absorbance (i.e., lipid peroxide levels) and independent variables = groups (with or without moxonidine) and time. The null hypothesis was rejected for two-sided *p* values of <0.05. All analyses were performed using SPSS version 27.0 (IBM SPSS Statistics for Windows, Armonk, NY, USA, IBM Corporation).

## Data Availability

Not applicable.

## Supplementary Materials

The following supporting information can be downloaded at: https://www.mdpi.com/article/10.3390/ijms24043857/s1.
