# Supplementary material for: Moxonidine Increases Uptake of Oxidised Low-Density Lipoprotein in Cultured Vascular Smooth Muscle Cells and Inhibits Atherosclerosis in Apolipoprotein E-Deficient Mice
Source: Int J Mol Sci. 2023 Feb 14;24(4):3857. doi: 10.3390/ijms24043857 (PMC9960795; doi:10.3390/ijms24043857)
Supplement: Supplementary file 1 [file ijms-24-03857-s001.zip › ijms-2095673-supplementary.pdf]

International Journal of
Molecular Sciences

MDPI

Supplementary figures and tables

Moxonidine Increases Uptake of Oxidised Low-Density Lipoprotein in Cultured Vascular Smooth Muscle Cells and Inhibits Atherosclerosis in Apolipoprotein E-Deficient Mice

Yutang Wang [1,*,†], Dinh Tam Nguyen [1,†], Jack Anesi [1], Ahmed Alramahi [1], Paul K. Witting [2], Zhonglin Chai [3], Abdul Waheed Khan [3], Jason Kelly [4], Kate M. Denton [5,6] and Jonathan Golledge [7,8]

[1] Discipline of Life Science, Institute of Innovation, Science and Sustainability, Federation University Australia, Ballarat, VIC 3350, Australia
[2] Molecular Biomedicine Theme, School of Medical Sciences, Faculty of Medicine and Health, Charles Perkins Centre, The University of Sydney, Sydney, NSW 2006, Australia
[3] Department of Diabetes, Central Clinical School, Monash University, Melbourne, VIC 3004, Australia
[4] Fiona Elsey Cancer Research Institute, Ballarat, VIC 3350, Australia
[5] Department of Physiology, Monash University, Melbourne, VIC 3800, Australia
[6] Cardiovascular Disease Program, Monash Biomedicine Discovery Institute, Monash University, Melbourne, VIC 3800, Australia
[7] Queensland Research Centre for Peripheral Vascular Disease, College of Medicine and Dentistry, James Cook University, Townsville, QLD 4811, Australia
[8] Department of Vascular and Endovascular Surgery, The Townsville University Hospital, Townsville, QLD 4814, Australia
* Correspondence: yutang.wang@federation.edu.au
† These authors contributed equally to this work.

Table S1: List of mouse primer sequences.

Name	Forward primer	Reverse primer
ABCA1	5′-CGTGTCTTGTCTGAAAAAGGAGG-3	5′-CGTGTCACTTTCATGGTCGC-3′
ABCG1	5′-GGCAGACGAGAGATGGTCAA-3′	5′-AAAGAACATGACAGGCGGGT-3′
EEF2	5’-ACATGTCAGTGATCGCCCAT-3’	5’-GAGATGGTGGTGGATTTGATTG-3’
IL-1	5’-GCCACCTTTTGACAGTGATGAA-3’	5’-GACAGCCCAGGTCAAAGGTT-3’
IL-6	5’-CGGCCTTCCCTACTTCACAA-3’	5’-GCCATTGCACAACTCTTTCTCA-3’
LDLR	5′-CCAATCGACTCACGGGTTCA-3′	5′-TCACACCAGTTCACCCCTCT-3′
MCP-1	5’-CTTCTGGGCCTGCTGTTCA-3’	5’-CGAGCCTACTCATTGGGATCA-3’
SR-B1	5′-GCTCGGCGTTGTCATGATCC-3′	5′-GGGTCTATGCGGACATTCTTGA-3′
TNF-α	5’-TAGCCCACGTCGTAGCAAAC-3’	5’-ACAAGGTACAACCCATCGGC-3’

Abbreviations: ABCA1, ATP binding cassette transporter A1; ABCG1, ATP binding cassette transporter G1; EEF-2, eukaryotic elongation factor 2; IL, interleukin; LDLR, low-density lipoprotein receptor; MCP-1, monocyte chemoattractant protein-1; SR-B1, Scavenger receptor class B type 1; TNF-*α*, tumour necrosis factor-α.

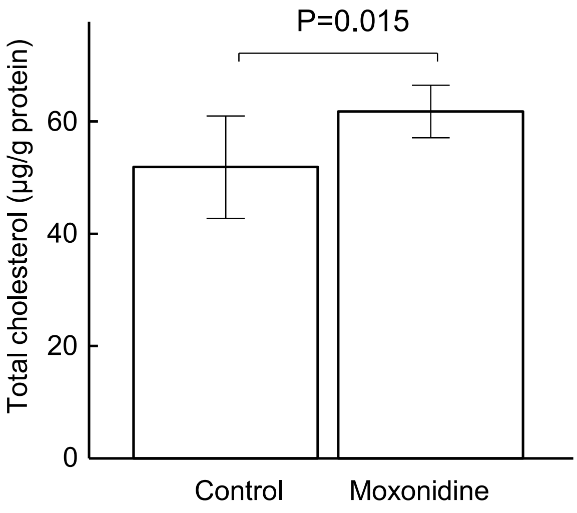

Figure S1: Effect of moxonidine on total cholesterol levels in VSMCs. VSMCs in 96-well plates were incubated with moxonidine (10 µM) or phosphate-buffered saline (PBS, control) for 2 h and the cells were then incubated with oxidised LDL (25 µg/L) for an additional 4h. The cells were washed and lysed, and the total cholesterol levels in the supernatant were measured. The difference was analysed by the Mann-Whitney U test. Error bar=SD; N=8. VSMCs, vascular smooth muscle cells.

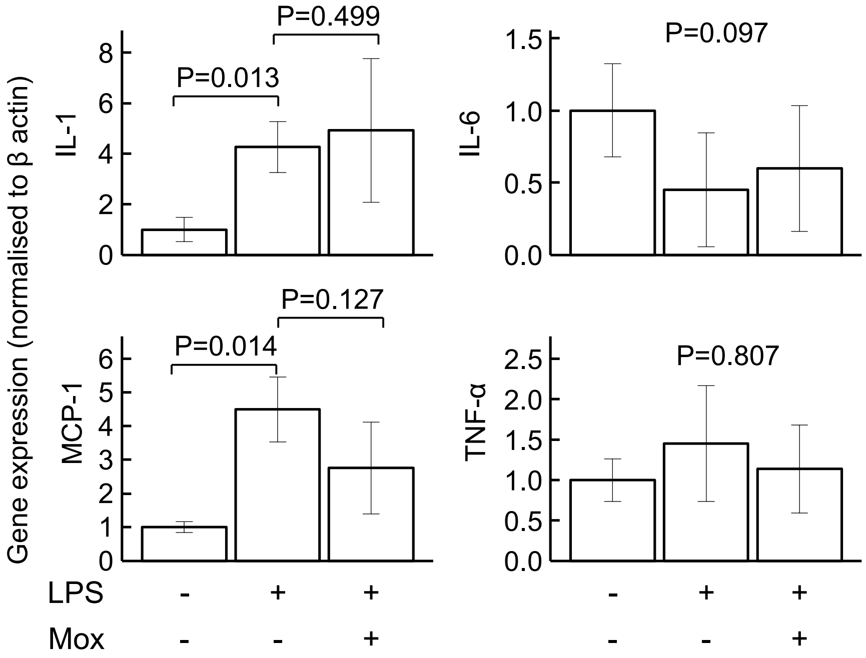

Figure S2: Effect of moxonidine on mRNA expression of inflammatory markers in human aortic endothelial cells. Cells were incubated with lipopolysaccharide (100 ng/mL) for 2 h in the absence or presence of moxonidine (1 μM). Then, mRNA was isolated and the expression of IL-1, IL-6, MCP-1 and TNF-α was quantified via quantitative PCR. The difference in gene expression was analysed by Kruskal-Wallis one-way ANOVA. Error bar=SE; N=5-6. IL, interleukin; LPS, lipopolysaccharide; MCP-1, monocyte chemoattractant protein-1; Mox, moxonidine; TNF-α, tumour necrosis factor α.

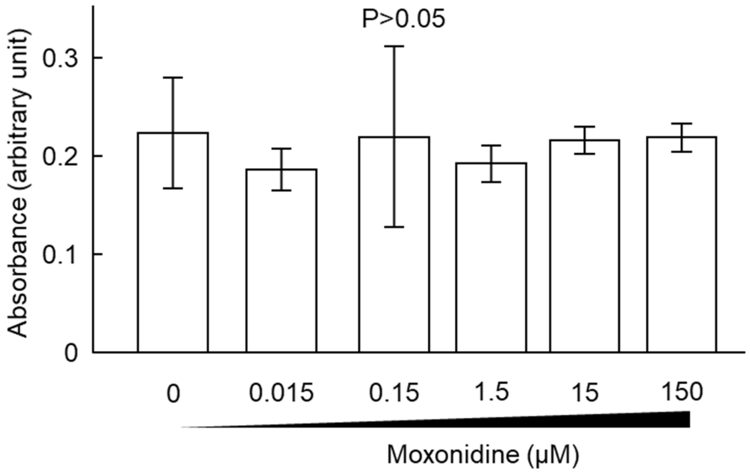

Figure S3: Effect of moxonidine on VSMC proliferation assessed by MTS assay. 200 μL of VSMCs (0.5 x 106 cells/ mL) were added to each well of a 96-well flat-bottom plate and kept at 37°C in an incubator overnight. Cells were then incubated with phosphate-buffered saline (PBS, control) or moxonidine (0.015, 0.15, 1.5, 15 and 150 μM) for 24h. The cell numbers were then estimated using the MTS assay. The difference among groups was analyzed using Kruskal-Wallis one-way ANOVA. Data represent mean ± SD; N=4. MTS, 3-(4,5-dimethylthiazol-2-yl)-5-(3-carboxymethoxyphenyl)-2-(4-sulfophenyl)-2H-tetrazolium; VSMC, vascular smooth muscle cell.

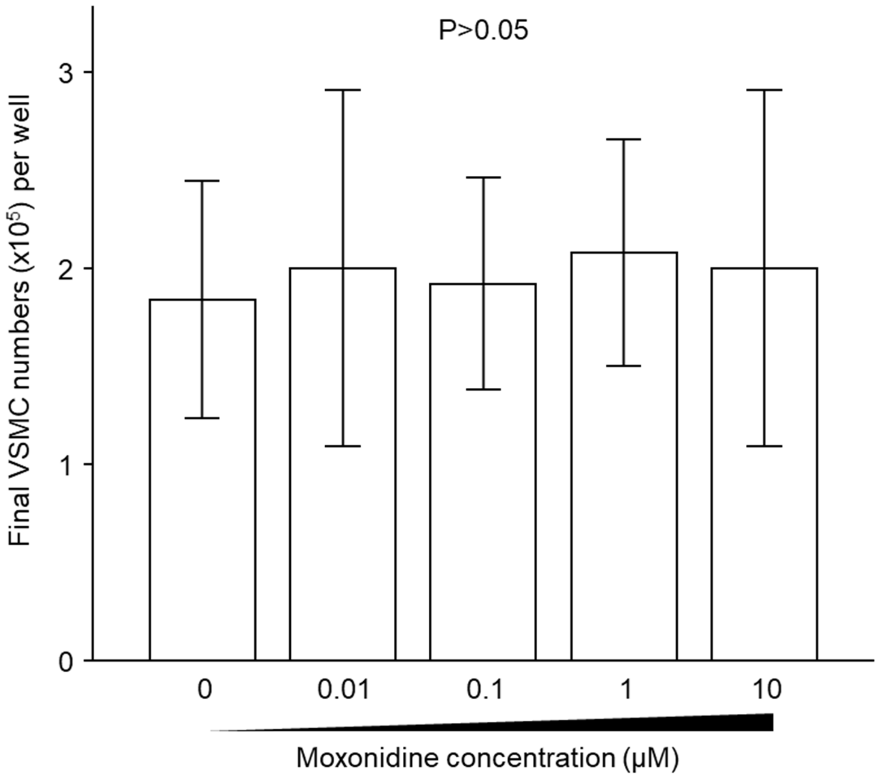

Figure S4: Effect of moxonidine on VSMC proliferation assessed by trypan blue method. VSMCs (1 x 105 cells) were placed in wells of 6-well plates and kept at 37°C in a 5% CO2 incubator for 24 h. Then the cells were incubated with moxonidine (final concentration, 0, 0.01, 0.1, 1, or 10 μM) for 24h. Then cell numbers were assessed using the trypan blue method. The difference among the groups was analysed by Kruskal-Wallis one-way AVOVA; data represent mean ± SD; N=4-5. VSMC, vascular smooth muscle cell.

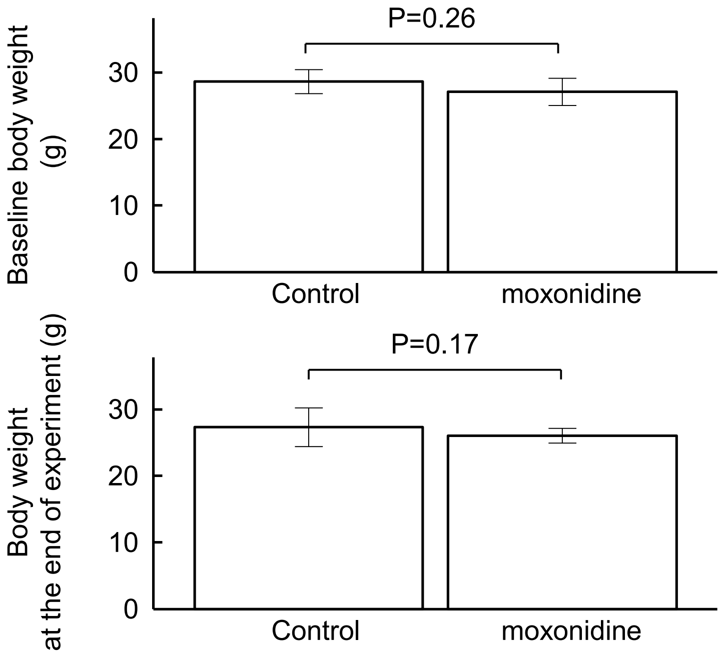

Figure S5: Body weight of the mice at the baseline and at the end of the experiment. N=6. Error bar=SD. The difference between the groups was analysed using the Mann-Whitney U test.

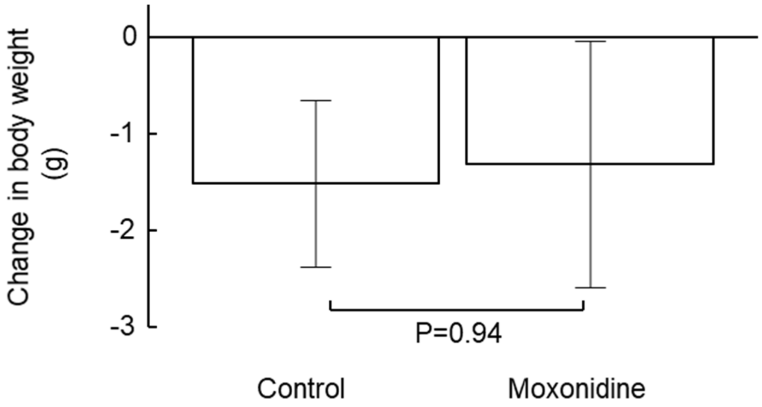

Figure S6: Chang in body weight of the mice during the experiment. N=6. Error bar=SD. The difference between the groups was analysed using the Mann-Whitney U test.

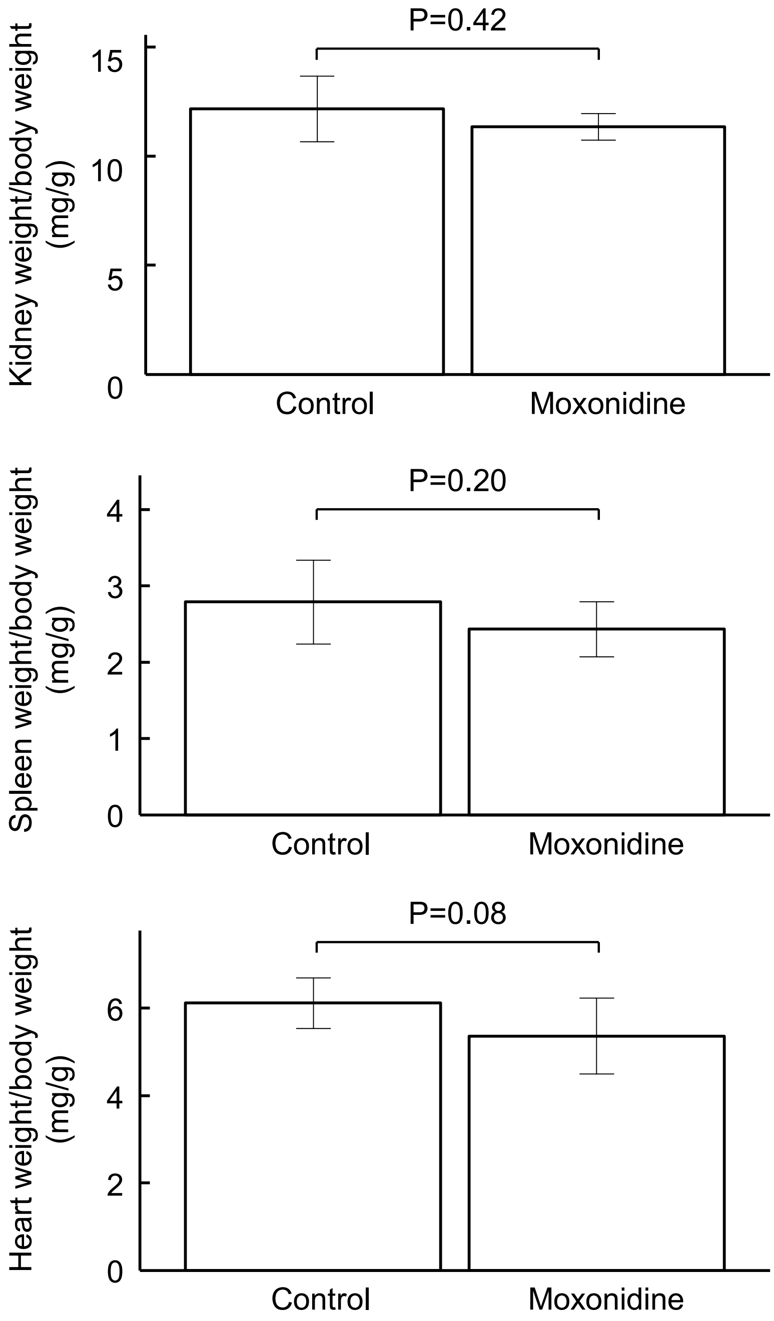

Figure S7: Organ weight of the mice at the end of the experiment. The weights of the kidney, spleen, and heart were expressed as the ratio of organ weight over body weight (mg/g). N=6. Error bar=SD. The difference between the groups was analysed using the Mann-Whitney U test.
